# Phytoremediation with *Geosiphon*-like symbiosis?

**DOI:** 10.1007/s11356-016-6135-1

**Published:** 2016-01-29

**Authors:** Grzegorz Wojtczak, Paulina Janik

**Affiliations:** Institute of Nature Conservation (Protection), Polish Academy of Sciences, Mickiewicza 33, Kraków, 31-120 Poland; W. Szafer Institute of Botany, Polish Academy of Sciences, Lubicz 46, Krakow, 31-512 Poland

Dear Editor,

In their recent article published in *Environmental Science and Pollution Research*, Anna Ogar et al. (2015) have shown, thanks to a careful experimental design, that the addition of diazotrophic bacteria together with mycorrhizal fungi significantly improves plant growth and performance. Strikingly, the addition of inoculum containing only diazotrophs but not mycorrhizal fungi had contrary effect and led to the reduced shoots and roots biomass and lower photosynthesis efficiency of *Medicago sativa* and *Hieracium pilosella*, as compared to experimental plants co-inoculated with mycorrhizal fungi and nitrogen-fixing bacteria. The authors gave their credit to many features of microbes used in the study that might have resulted in such a visible change. Reasons for growth inhibition by diazotrophic bacteria not accompanied by mycorrhizae seem well justified. However, positive impact of combined addition of mycorrhizae and diazotrophs deserves, in our opinion, examining of another relevant explanation.

As hypothesized by Pirozynski and Malloch (1975) and evidenced later by many (Selosse and Le Tacon 1998; Smith and Read 2008), mycorrhizal fungi enabled plants’ ancestors to conquer terrestrial habitat. It is also more and more often suggested, that mycorrhizal fungi led to the evolution of roots themselves, in a similar way symbiotic microbes drove the evolution of animals’ gut (as argued by Margaret McFall-Ngai in Velasquez-Manoff 2015). The comparison between root and gut is not a simple analogy, as in both cases symbiotic microbes significantly contribute to nutrition and protection of their hosts, and several developmental processes are mirrored in those seemingly different organs making their mode of evolution follow the same pattern (Selosse et al. 2014). *Rhizophagus irregularis* (syn. *Glomus intraradices*), an arbuscular mycorrhizal fungus (phylum *Glomeromycota*), was used by Ogar and her co-workers. *Glomus sensu lato* descended from the hypothetical, primordial-plant mycorrhizal symbionts and as all members of this group are unable to synthesize simple sugars, nor to take them up from the environment. For this, symbiosis with appropriate photosynthetic partner(s) needs to be established. Despite such dependency, scientists are quite sure that the presence of mycorrhizal fungi in soils preceded and was a prerequisite for green plants’ “invasion” (Redecker et al. 2000). While occupying land before green plants’ emergence, mycorrhizal fungi must have been involved in associations with other phototrophs. One of the *Glomeromycota* members does so until nowadays: *Geosiphon pyriforme* is a unique microorganism that has a rather stormy history of taxonomical record. First described as an alga, it is now known to be a truly *Glomeromycota* and the only fungus known to endocytobiotically host cyanobacteria (Schüßler 2012). *Geosiphon* associates with various members of cyanobacterial order *Nostocales*. However, it may also form symbiosis with plants (Schüßler 2012). Accordingly, the existence of interaction between other *Glomeromycota* with cyanobacteria, just like *Geosiphon*, cannot be ruled out.

Quite recently, physically and metabolically close associations have been artificially induced between unicellular green alga—*Chlamydomonas* and a hyphae of a mold-like, “non-symbiotic” fungus—*Aspergillus*, as well as between *Chlamydomonas* and yeasts (Hom and Murray 2014). This phenomenon was suggested to prove latent capacity for mutualistic symbiosis in non-symbiotic microorganisms (Aanen and Bisseling 2014; Hom and Murray 2014). It is therefore very likely, that “beads on a string”–like organization might have appeared also in reference to *Rhizophagus* hyphae and diazotrophic bacteria used in the study performed by Anna Ogar. This is supported by a fact that *Nostoc* strains were used as one of two diazotrophic taxa added. Higher net plant biomass production and better photosynthesis performance could be easily explained by direct interactions between cyanobacterium and mycorrhizal fungus. The latter being provided with photosynthates from prokaryote would be less reliable on nutrients transferred from the plant host. This, on the other hand, could allow saving significant amount of carbon compounds assimilated by plants and the nitrogen provided by cyanobacterium could be much more accessible for the plant in the presence of fungal mediation than in its absence. Our view is also in accordance with the recently flourishing research on hyphosphere (the soil surrounding the hyphae) and hyphospheric bacteria, present in the vicinity of fungal hyphae. In case of *Glomeromycota*, specific unique species-strain compositions of microbes are being described as well as their important role in the functioning of mycorrhizal symbiosis (Puppi et al. 1994; Scheublin et al. 2010). For example, particular phosphate solubilizing bacteria associated with *Glomeromycota* hyphae may directly influence the phosphorus turnover and the effective usage of its resources is highly enhanced by interactions between *Glomeromycota* and hyphospheric bacteria (Zhang et al. 2014). Interactions in the hyphosphere also shape nitrogen cycling, with significant contribution from archea—largely extending taxonomical scope of these communities (Chen et al. 2013).

The research led by Anna Ogar was a part of a long-term studies devoted to the reclamation of heavy metal-rich industrial wastes, initiated more than 25 years ago (Turnau et al. 2012), and the soil-substratum used in the experiment originated from the reclaimed Zn-Pb tailing. Because heavy metal-tailing substratum is for many reasons harsh and suppressive for plant growth (Turnau et al. 2012; Ogar et al. 2015), the conditions in which plants had been brought up were challenging in a similar way as during primary succession. One could even go further with such reasoning and suggest that they were similar to those faced by primary plants in their primordial environment, in more ancient time. This would, at least to some extent, mirror conditions in which *Geosiphon* symbiosis evolved and could favor expression of hypothetical ancient genetic program, enabling interaction with cyanobacteria. Positive impact of inoculum containing both diazotrophs and mycorrhizae was confirmed on dry heat sterilized substratum. This allows exclusion of involvement from indigenous microbes and makes almost certain that the interactions between added microbes were responsible for such significant change in plants’ growth and performance. Also, the particular *Nostoc* strain used in the experiment, *N. edaphicum*, originated from the same tailing substratum and was previously described to be adapted to grow in such conditions (Ogar et al. 2015). Thus, there is little doubt that it might not survive in the course of experiment. Still, however, the above statements will remain speculative as long as not examined by researchers.

An interesting task challenging our model would be tracing for presence of suggested, intimate interactions between diazotrophic cyanobacterium and mycorrhizae. Indeed, it would be a noteworthy approach allowing also complete fulfillment of Koch’s postulates. The authors have already accomplished that by standard methodology of root staining while evaluation of mycorrhizal colonization parameters. Examination of fresh substratum samples in the search of cyanobacteria and with the use of light microscopy might successfully supplement these data. It could also reveal presence of associations between hyphae of *Glomeromycota* and cyanobacterium. Unfortunately, this may not be easy to perform, since hyphosphere is much more difficult to access with standard procedures (Scheublin et al. 2010). However, once developed by researchers, new approaches might easily become an example of basic research with immediate environmental implementation. Thick on the ground presence of such cyanobacteria, altogether with findings mentioned by Ogar et al., could lead to a new insight into already applied restoration practices on heavy metal-rich, postindustrial areas and pave the way to novel practices, largely improving revegetation on such sites in the nearest future.
